# Supplementary material for: MetaRibo-Seq measures translation in microbiomes
Source: Nat Commun. 2020 Jun 29;11:3268. doi: 10.1038/s41467-020-17081-z (PMC7324362; doi:10.1038/s41467-020-17081-z)
Supplement: Supplementary file 10 — Supplementary Data 7 [file 41467_2020_17081_MOESM10_ESM.zip › File2/Confidence_High_Taxonomy/333711_out.krona.html]

Javascript must be enabled to view this page.

members
magnitude
magnitudeUnassigned
count
unassigned
taxon
rank

333711\_out

34

2
34
superkingdom

1239
phylum
33

186801
class
33

order
33
186802

1950935

SRS013965\_contig\_number\_4561SRS024663\_contig\_number\_7192SRS101433\_contig\_number\_3637
species
3


SRS013098\_contig\_number\_contig-100\_3660.236775SRS013940\_contig\_number\_13921SRS014613\_contig\_number\_16505SRS014855\_contig\_number\_1587SRS017307\_contig\_number\_29628SRS018351\_contig\_number\_contig-100\_7091.70373SRS018936\_contig\_number\_contig-100\_1637.48735SRS047433\_contig\_number\_9385SRS050998\_contig\_number\_12827SRS053356\_contig\_number\_44932SRS063518\_contig\_number\_30092SRS064276\_contig\_number\_contig-100\_7010.176522SRS075078\_contig\_number\_9840SRS077194\_contig\_number\_23259SRS077294\_contig\_number\_4908SRS098644\_contig\_number\_27672SRS1041091\_contig\_number\_8898SRS104311\_contig\_number\_41969SRS104636\_contig\_number\_27009SRS1054691\_contig\_number\_contig-100\_5495.92987SRS140492\_contig\_number\_14057SRS142890\_contig\_number\_25276SRS144297\_contig\_number\_12691SRS144714\_contig\_number\_15753SRS147139\_contig\_number\_contig-100\_3570.216212SRS148159\_contig\_number\_44658SRS148721\_contig\_number\_30380SRS893373\_contig\_number\_1839
1898207
28
species

2
family
541000

1
genus
216851


SRS015264\_contig\_number\_contig-100\_3628.40035
853
species
1


SRS049896\_contig\_number\_23896
1898205
1
species

976
1
phylum

class
1
200643

171549
1
order

171550
family
1


SRS044535\_contig\_number\_969
239759
1
genus
